# Early changes in laboratory tests predict liver function damage in patients with moderate coronavirus disease 2019: a retrospective multicenter study

**DOI:** 10.1186/s12876-022-02188-y

**Published:** 2022-03-09

**Authors:** Yiting Wang, Dandan Gao, Xuewen Li, Panyang Xu, Qi Zhou, Junguo Yin, Jiancheng Xu

**Affiliations:** 1 Department of Laboratory Medicine, First Hospital of Jilin University, 1 Xinmin Street, Changchun, 130021 China; 2 Department of Pediatrics, First Hospital of Jilin University, Changchun, 130021 China; 3 Department of Laboratory Medicine, Changchun Hospital of Traditional Chinese Medicine, Changchun, 130021 China

**Keywords:** Coronavirus disease 2019, Liver function, Plateletcrit, Retinol-binding protein, Carbon dioxide combining power, Nomogram, Predict

## Abstract

**Background:**

Most patients with coronavirus disease 2019 demonstrate liver function damage. In this study, the laboratory test data of patients with moderate coronavirus disease 2019 were used to establish and evaluate an early prediction model to assess the risk of liver function damage.

**Methods:**

Clinical data and the first laboratory examination results of 101 patients with moderate coronavirus disease 2019 were collected from four hospitals’ electronic medical record systems in Jilin Province, China. Data were randomly divided into training and validation sets. A logistic regression analysis was used to determine the independent factors related to liver function damage in patients in the training set to establish a prediction model. Model discrimination, calibration, and clinical usefulness were evaluated in the training and validation sets.

**Results:**

The logistic regression analysis showed that plateletcrit, retinol-binding protein, and carbon dioxide combining power could predict liver function damage (*P* < 0.05 for all). The receiver operating characteristic curve showed high model discrimination (training set area under the curve: 0.899, validation set area under the curve: 0.800; *P* < 0.05). The calibration curve showed a good fit (training set:* P* = 0.59, validation set:* P* = 0.19; *P* > 0.05). A decision curve analysis confirmed the clinical usefulness of this model.

**Conclusions:**

In this study, the combined model assesses liver function damage in patients with moderate coronavirus disease 2019 performed well. Thus, it may be helpful as a reference for clinical differentiation of liver function damage.

*Trial registration* retrospectively registered.

## Background

Severe acute respiratory syndrome coronavirus 2 was first detected in December 2019, and the World Health Organization named the disease coronavirus disease 2019 (COVID-19) in February 2020 [1]. COVID-19 has become a global public health problem. As of January 10, 2022, there were over 300 million confirmed cases of COVID-19 worldwide and over 5.48 million deaths [2]. Studies have shown that COVID-19 can affect lung [3], kidney [4], liver [5], heart [6], and gastrointestinal [7] function, which can be life-threatening if left untreated.

The liver is an essential organ in the human body, and its condition can be assessed using a combination of examinations. One study [8] has shown that liver function damage is closely associated with a poor prognosis in patients with COVID-19. However, the specific mechanism of liver function damage is still unclear, but it may be due to multiple factors. First, the virus directly infects hepatocytes or combines with bile duct cells to cause bile duct dysfunction, leading to liver function damage [9]. Second, the cytokine storm and systemic inflammatory response syndrome occur in the body, leading to liver function damage [10]. Third, drug interactions during treatment may damage liver function [11]. Finally, patients with hypoxemia and respiratory distress syndrome exhibit an oxidative stress response, leading to liver function damage [12]. At present, most patients with COVID-19 have mild symptoms, and the clinical manifestations of early liver dysfunction are not obvious [13]. If doctors cannot detect liver dysfunction, patients cannot be treated promptly, and their disease worsens. We conducted this retrospective multicenter study in Jilin Province, China. The study aimed to identify indicators related to liver function damage in patients with moderate COVID-19 and establish an early prediction model for liver function damage.

## Methods

### Study design and patients

A confirmed case of COVID-19 was defined as a positive real-time reverse-transcriptase-polymerase-chain-reaction assay result from sputum and throat swab specimens [14]. The patients with mild, moderate, severe, and critical COVID-19 were diagnosed by the Diagnosis and treatment protocol for novel coronavirus pneumonia (Trial Version 7) [15]. Patients with mild COVID‑19 had mild clinical symptoms and no pneumonia signs on imaging. Moderate cases had a fever and respiratory symptoms with imaging findings of pneumonia. Cases meeting any of the following criteria were defined as severe cases: Respiratory distress (respiratory rate, ≥ 30 breaths/min); oxygen saturation ≤ 93% at rest; arterial oxygen partial pressure/fraction of inspired oxygen ≤ 300 mmHg. Lung imaging indicated that the lesions progressed significantly within 24–48 h, and patients with lung lesions occupying > 50% of the lung were treated according to management protocols for severe cases. Cases meeting any of the following criteria were defined as critical cases: Respiratory failure and requirement of mechanical ventilation; shock; combination with failure of other organs that required care at the intensive care unit. One hundred one patients admitted to the isolation wards of the hospital diagnosed with moderate COVID-19 were included in this study. None of the patients had a history of liver disease and malnutrition. The data were collected in the electronic medical record systems of the First Hospital of Jilin University, Changchun Infectious Diseases Hospital, Changchun Chinese Medicine Hospital and Siping Infectious Diseases Hospital from January 2020 to March 2021. All the patients were discharged at the time of collection. Data included sex, age, comorbidities, chief complaint, length of hospitalization, and the results of the first laboratory examination after admission. The four hospitals were all designated hospitals for COVID-19 treatment in Jilin province. Data were randomly divided into a training set and a validation set at a ratio of 7:3. The training set was used to build the model, while the validation set was used for internal confirmation. Patients were divided into four subgroups based on liver function. The study was conducted in accordance with the Declaration of Helsinki (as revised in 2013). The ethics committees approved this study of the First Hospital of Jilin University (No. 2020-313), Changchun Infectious Disease Hospital (No. 2020-001), Changchun Hospital of Traditional Chinese Medicine (No. 2021-005), and Siping Infectious Disease Hospital (No. 2020-001). The requirement for written informed consent was waived owing to the retrospective nature of the study by the ethics committees.

### Data collection

Laboratory tests included hematological and biochemical tests. Some laboratory data were absent due to a lack of specific test results. The blood and biochemical equipment used at the First Hospital of Jilin University were XN-9000 (Sysmex Corp., Hyogo, Japan), CS-5100 (Sysmex Corp.), and 7600-210 (Hitachi High-Technologies, Tokyo, Japan). The blood and biochemical equipment used at Changchun Infectious Disease Hospital were DF53 (Dymind Biotechnology Corp., Shenzhen, China), OCG-102 (Wondfo Biotech Corp., Guangzhou, China), and CS-T300 (Dirui Industrial Corp., Changchun, China). The blood and biochemical equipment used at Changchun Chinese Medicine Hospital were BC-5390 (Mindray Biomedical Electronics Corp., Shenzhen, China) and B-S800M (Mindray Biomedical Electronics Corp., Shenzhen, China). The blood and biochemical equipment used at Siping Infectious Disease Hospital were ABX Pentra XL 80 (Horiba Medical, Montpellier, France), CS-2500 (Sysmex Corp.), and Pointcare M3i (Mnchip Technology Corp., Tianjin, China). These four laboratories passed the external quality assessment and capability certification of Jilin Provincial Clinical Laboratory Center. The instruments were tested by strict quality control before use. Test kits, calibrators, and quality control products were the same at all four hospitals. Liver function damage was defined when one or more indicators listed in Table 1 exceeded 1.5 times of the upper limit, or total protein, albumin was lower than 0.5 times of the lower limit.

**Table 1:** Diagnostic criteria for liver function damage

Indicator	Reference intervals	
	Male	Female
TP (g/L)	65–85	
ALB (g/L)	40–55	
ALT (U/L)	9–60	7–45
AST (U/L)	15–45	13–40
ALP (U/L)	45–125	35–100 (< 50 years)
		50–135 (≥ 50 years)
GGT (U/L)	10–60	7–45
TBIL (μmol/L)	0–26	0–21

TP, total protein; ALB, albumin; ALT, alanine aminotransferase; AST, aspartate aminotransferase; ALP, alkaline phosphatase; GGT, γ-glutamyl transpeptidase; TBIL, total bilirubin

### Statistical analysis

Categorical variables were compared using the chi-square test or Fisher’s exact test and represented by *n* (frequency). Continuous variables with a normal distribution were compared using the *t-*test and are characterized by average. Continuous variables with a non-normal distribution were compared using the Mann–Whitney *U* test and are represented by median. Spearman’s correlation coefficient was used for correlation analysis. Univariate and multivariate logistic regression analyses analyzed the independent factors indicating liver function damage in patients with moderate COVID-19, and odds ratios (ORs) and 95% confidence intervals (CIs) were calculated. A nomogram was constructed based on these independent factors. Prediction model discrimination was evaluated using the receiver operating characteristic (ROC) curve. The area under the ROC curve (AUC) and 95% CI were calculated. An AUC of > 0.75 was considered to indicate good model performance. A* P* value of < 0.05 was considered statistically significant. The calibration curve was used to evaluate the model’s goodness of fit. A *P* value of > 0.05 was considered a satisfactory fit. A decision curve analysis (DCA) was used to evaluate the clinical usefulness of the model. Stata 15.0 (Stata Corp LLC, Texas, USA) and GraphPad Prism 8.0 (GraphPad Software Corp, San Diego, USA) software were used for data analysis.

## Results

### Clinical and laboratory characteristics of patients with moderate COVID-19 on admission

A total of 101 patients with moderate COVID-19 were randomly divided into a training set (*n* = 70) and a validation set (*n* = 31) at a ratio of 7:3. A liver function damage group (*n* = 45) and a normal liver function group (*n* = 25) comprised the training set. Similarly, a liver function damage group (*n* = 21) and a normal liver function group (*n* = 10) comprised the validation set. In terms of the training set in general, the average age of patients was 52.81 years, 48.57% of patients were male, the average length of hospitalization was 19.69 days, and the most common complications were cardiovascular (25.71%) and endocrine system (11.43%) diseases. There were no significant differences in sex, age, and length of hospitalization between the liver function damage group and the normal liver function group (*P* < 0.05). In the training set, alkaline phosphatase, creatinine, carbon dioxide combining power (CO_2_-CP), retinol-binding protein (RBP), platelet distribution width, plateletcrit (PCT), mean platelet volume, red blood cell count, hematocrit, hemoglobin, white blood cell (WBC) count, neutrophil percentage (NE%), lymphocyte count, lymphocyte percentage, monocyte percentage (MO%), thrombin time, prothrombin time (PT), sodium, potassium, chloride (Cl), calcium (Ca), cholesterol, high-density lipoprotein cholesterol (HDL-C), and low-density lipoprotein cholesterol data were normally distributed. Total protein, albumin, alanine aminotransferase, aspartate aminotransferase, γ-glutamyl transpeptidase, total bilirubin, CO_2_-CP, RBP, PCT, MO%, PT, Cl, Ca, HDL-C, high-sensitivity C-reactive protein (hsCRP), and lactate dehydrogenase (LDH) were significantly different between the liver function damage group and the normal liver function group (*t* or *Z* values were − 2.605, − 2.409, 2.306, 3.234, 5.990, − 2.673, − 2.656, − 2.462, − 2.917, − 2.332, − 2.550, − 2.231, − 4.227, − 2.212, 1.977, and 2.824, respectively; *P* values were 0.009, 0.016, 0.021, 0.001, 0.001, 0.008, 0.010, 0.017, 0.005, 0.023, 0.014, 0.029, 0.001, 0.031, 0.048, and 0.005, respectively). Patients’ clinical characteristics and the results of the first laboratory examination after admission are summarized in Tables 2 and 3.

**Table 2:** Clinical characteristics of patients with moderate COVID-19 on admission

Characteristics	Training set (*n* = 70)			Validation set (*n* = 31)		
	All patients (*n* = 70)	Liver function damage group (*n* = 45)	Normal liver function group (*n* = 25)	All patients (*n* = 31)	Liver function damage group (*n* = 21)	Normal liver function group (*n* = 10)
*Sex n (%)*						
Male	34 (48.6)	22 (48.9)	12 (48.0)	14 (45.2)	8 (38.1)	6 (60.0)
Female	36 (51.4)	23 (51.1)	13 (52.0)	17 (54.8)	13 (61.9)	4 (40.0)
Age (years) $\overline{x }\pm s$/*M* (*IQR*)	52.81 ± 15.92	54.11 ± 15.77	50.48 ± 16.24	52.06 ± 18.52	56.14 ± 18.41	43.50 ± 16.44
*Any comorbidities n (%)*						
Cardiovascular disease	18 (25.7)	14 (31.1)	4 (16.0)	5 (16.1)	4 (19.1)	1 (10.0)
Endocrine system disease	8 (11.4)	4 (8.9)	4 (16.0)	4 (12.9)	4 (19.1)	-
Others	11 (15.7)	9 (20.0)	2 (8.0)	5 (16.1)	4 (19.1)	1 (10.0)
*Chief complaint n (%)*						
Cough	34 (48.6)	27 (60.0)*	7 (28.0)	20 (64.5)	14 (66.7)	6 (60.0)
Fever	21 (30.0)	15 (33.3)	6 (24.0)	11 (35.5)	10 (47.6)	1 (10.0)
Fatigue	9 (12.7)	6 (13.3)	3 (12.0)	7 (22.6)	4 (19.1)	3 (30.0)
Others	11 (15.7)	8 (17.8)	3 (12.0)	10 (32.3)	7 (33.3)	3 (30.0)
*Drug use n (%)*						
Traditional Chinese medicine	59 (84.3)	39 (86.7)	20 (80.0)	23 (74.2)	17 (81.0)	6 (60.0)
Antiviral drugs	54 (77.1)	32 (71.1)	22 (88.0)	19 (61.3)	13 (61.9)	6 (60.0)
Anti-inflammatory drugs	12 (17.1)	8 (17.8)	4 (16.0)	2 (6.5)	2 (9.5)	–
Others	16 (22.9)	12 (26.7)	4 (16.0)	1 (3.2)	–	1 (10.0)
Length of hospitalization (days) $\overline{x }\pm s$/*M* (*IQR*)	19.69 ± 6.90	19.42 ± 7.60	20.16 ± 5.54	20.00 (16.00–24.00)	20.00 (17.00–24.00)	16.50 (11.75–21.25)

COVID-19: coronavirus disease 2019. $\overline{x }$: Average. *s*: Standard deviation. *M* (*IQR*): Median (Quartile)

*Significant difference between the liver function damage group and the normal liver function group, *P* < 0.05

**Table 3:** Laboratory characteristics of patients with moderate COVID-19 on admission

Analytes	Training set (*n* = 70)						Validation set (*n* = 31)					
	All patients		Liver function damage group		Normal liver function group		All patients		Liver function damage group		Normal liver function group	
	*n*	***M***/$\overline{x }$	*n*	***M***/$\overline{x }$	*n*	***M***/$\overline{x }$	*n*	***M***/$\overline{x }$	*n*	***M***/$\overline{x }$	*n*	***M***/$\overline{x }$
*Liver function parameters*												
TP	70	**69.50**	45	69.22*	25	**71.25**	22	**68.71**	12	65.58*	10	**73.14**
ALB	70	**40.49**	45	38.15*	25	**42.00**	22	39.55	12	35.94*	10	43.87
ALT	70	**25.00**	45	31.00*	25	**19.00**	31	**19.70**	21	16.00*	10	**24.00**
AST	70	**26.00**	45	28.00*	25	**21.00**	31	**22.00**	21	**22.00**	10	**23.50**
ALP	59	70.73	34	68.32	25	74.00	22	60.87	12	60.51	10	61.30
GGT	70	**59.00**	45	75.00*	25	**19.00**	31	**68.00**	21	70.00*	10	**24.50**
TBIL	70	**9.50**	45	8.30*	25	**11.40**	31	**9.60**	21	8.00*	10	**14.50**
*Kidney function parameters*												
Glu	70	**6.36**	45	**6.39**	25	**6.17**	31	**6.17**	21	**6.59**	10	**5.65**
Cr	70	67.35	45	67.21	25	67.61	31	65.69	21	65.00	10	67.14
Ur	70	**3.69**	45	**3.65**	25	**4.02**	31	**4.17**	21	**4.17**	10	**3.96**
CO_2_-CP	70	24.49	45	23.93*	25	25.48	31	24.37	21	22.83*	10	26.90
UA	54	**279.25**	32	**238.05**	22	**290.50**	15	259.63	11	259.19	4	260.83
RBP	53	31.55	31	28.72*	22	35.5	15	29.64	11	27.91	4	34.41
*Platelet parameters*												
PLT	70	**189.50**	45	**188.00**	25	**194.00**	31	186.84	21	179.10	10	203.10
PDW	70	12.35	45	12.11	25	12.77	31	13.24	21	12.37*	10	15.06
PCT	48	12.48	26	10.94*	22	14.29	27	13.36	17	13.22	10	13.59
MPV	70	10.38	45	10.35	25	10.43	31	9.96	21	10.0	10	9.7
*Erythrocyte parameters*												
RBC	70	4.53	45	4.48	25	4.63	31	4.44	21	4.27*	10	4.81
HCT	70	0.41	45	0.40	25	0.41	31	0.40	21	0.39*	10	0.43
HGB	70	139.05	45	138.24	25	140.52	31	138.07	21	132.90*	10	148.92
MCV	70	**89.50**	45	**89.60**	25	**89.50**	31	90.77	21	90.68	10	90.96
MCH	70	**30.50**	45	**30.50**	25	**30.50**	31	31.14	21	31.13	10	31.16
MCHC	70	**342.00**	45	**342.00**	25	**341.00**	31	343.32	21	343.57	10	342.80
RDW	70	**11.95**	45	**11.90**	25	**12.10**	31	11.9	21	12.08	10	11.69
*Leukocyte parameters*												
WBC	70	5.51	45	5.69	25	5.18	31	5.01	21	5.04	10	4.93
NE#	70	**3.31**	45	**3.40**	25	**2.60**	31	3.19	21	3.16	10	3.25
NE%	70	61.73	45	62.49	25	60.37	31	**62.40**	21	**63.10**	10	**61.60**
LY#	70	1.46	45	1.46	25	1.44	31	1.33	21	1.39	10	1.19
LY%	70	28.01	45	27.80	25	28.40	31	**27.30**	21	**27.60**	10	**23.95**
MO#	70	**0.40**	45	**0.40**	25	**0.50**	31	**0.50**	21	**0.32**	10	**0.50**
MO%	70	9.02	45	8.45*	25	10.06	31	8.42	21	7.58*	10	10.20
*Coagulation parameters*												
APTT	47	**32.70**	28	**32.75**	19	**32.70**	17	**35.80**	14	**35.95**	3	**33.70**
TT	47	15.15	28	15.08	19	15.26	17	15.49	14	15.61	3	14.93
PT	47	13.37	28	13.04*	19	13.86	17	12.64	14	12.46	3	13.47
INR	47	**1.03**	28	**1.02**	19	**1.05**	17	1.04	14	1.04	3	1.03
FBG	47	**3.03**	28	**3.21**	19	**2.86**	17	3.13	14	3.06	3	3.48
*Electrolyte parameters*												
Na	64	139.51	41	139.13	23	140.17	28	139.05	19	139.82	9	137.43
K	64	4.02	41	4.03	23	4.00	28	4.05	19	4.00	9	4.17
Cl	64	102.12	41	101.42*	23	103.35	28	100.58	19	101.04	9	99.61
Ca	48	9.82	28	9.62*	20	10.09	13	**9.58**	10	**9.44**	3	**9.98**
*Blood lipid parameters*												
TG	55	**1.16**	33	**1.19**	22	**1.15**	15	**1.01**	11	**1.05**	4	**0.97**
CHO	55	3.91	33	3.79	22	4.09	15	3.78	11	3.76	4	3.84
HDL-C	55	0.84	33	0.80*	22	0.90	15	0.85	11	0.85	4	0.86
LDL-C	54	2.00	32	1.96	22	2.06	15	1.90	11	1.91	4	1.88
*Cardiac markers parameters*												
hsCRP	52	**8.25**	31	14.70*	21	**6.01**	24	**11.92**	17	**20.79**	7	**6.00**
CK	42	**78.00**	26	**90.00**	16	**72.50**	25	**66.00**	16	**65.50**	9	**67.00**
CK-MB	56	**13.00**	35	**14.00**	21	**12.00**	29	**12.00**	19	**13.00**	10	**12.00**
LDH	42	**193.00**	26	206.00*	16	**182.00**	25	213.24	16	216.81	9	206.89

TP, Total protein, g/L; ALB, Albumin, g/L; ALT, Alanine aminotransferase, U/L; AST, Aspartate aminotransferase, U/L; ALP, Alkaline phosphatase, U/L; GGT, γ-glutamyl transpeptidase, U/L; TBIL, Total bilirubin, μmol/L; Glu, Glucose, mol/L; Cr, Creatinine, μmol/L; Ur, Urea, mmol/L; CO_2_-CP, Carbon dioxide combining power, mmol/L; UA, Uric acid, μmol/L; RBP, Retinol-binding protein, mg/L; PLT, Platelet count, × 10^9^/L; PDW, Platelet distribution width, %; PCT, Plateletcrit, ‱; MPV, Mean platelet volume, fL; RBC, Red blood cell, × 10^12^/L; HCT, Hematocrit, L/L; HGB, Hemoglobin, g/L; MCV, Mean corpuscular volume, fL; MCH, Mean corpuscular hemoglobin, pg; MCHC, Mean corpuscular hemoglobin concentration, g/L; RDW, Red blood cell distribution width, %; WBC, White blood cell, × 10^9^/L; NE#, Neutrophil count, × 10^9^/L; NE%, Neutrophil percentage, %; LY#, Lymphocyte count, × 10^9^/L; LY%, Lymphocyte percentage, %; MO#, Monocyte count, × 10^9^/L; MO%, Monocyte percentage, %; APTT, Activated partial thromboplastin time, s; TT, Thrombin time, s; PT, Prothrombin time, s; INR, International normalized ratio; FBG, Fibrinogen, g/L; Na, Sodium, mmol/L; K, Potassium, mmol/L; Cl, Chloride, mmol/L; Ca, Calcium, mg/dL; TG, Triglyceride, mmol/L; CHO, Cholesterol, mmol/L; HDL-C, High-density lipoprotein cholesterol, mmol/L; LDL-C, Low-density lipoprotein cholesterol, mmol/L; hsCRP, High-sensitivity C-reactive protein, mg/L; CK, Creatine kinase, U/L; CK-MB, Creatinine kinase-muscle/brain activity, U/L; LDH, Lactate dehydrogenase, U/L. COVID-19: coronavirus disease 2019.$\overline{x }$: Average. *M*: Median. The bold fonts represent the median

*: Significant difference between the liver function damage group and the normal liver function group, *P* < 0.05

### Establishing a model to predict liver function damage in patients with moderate COVID-19

**Fig. 1:**
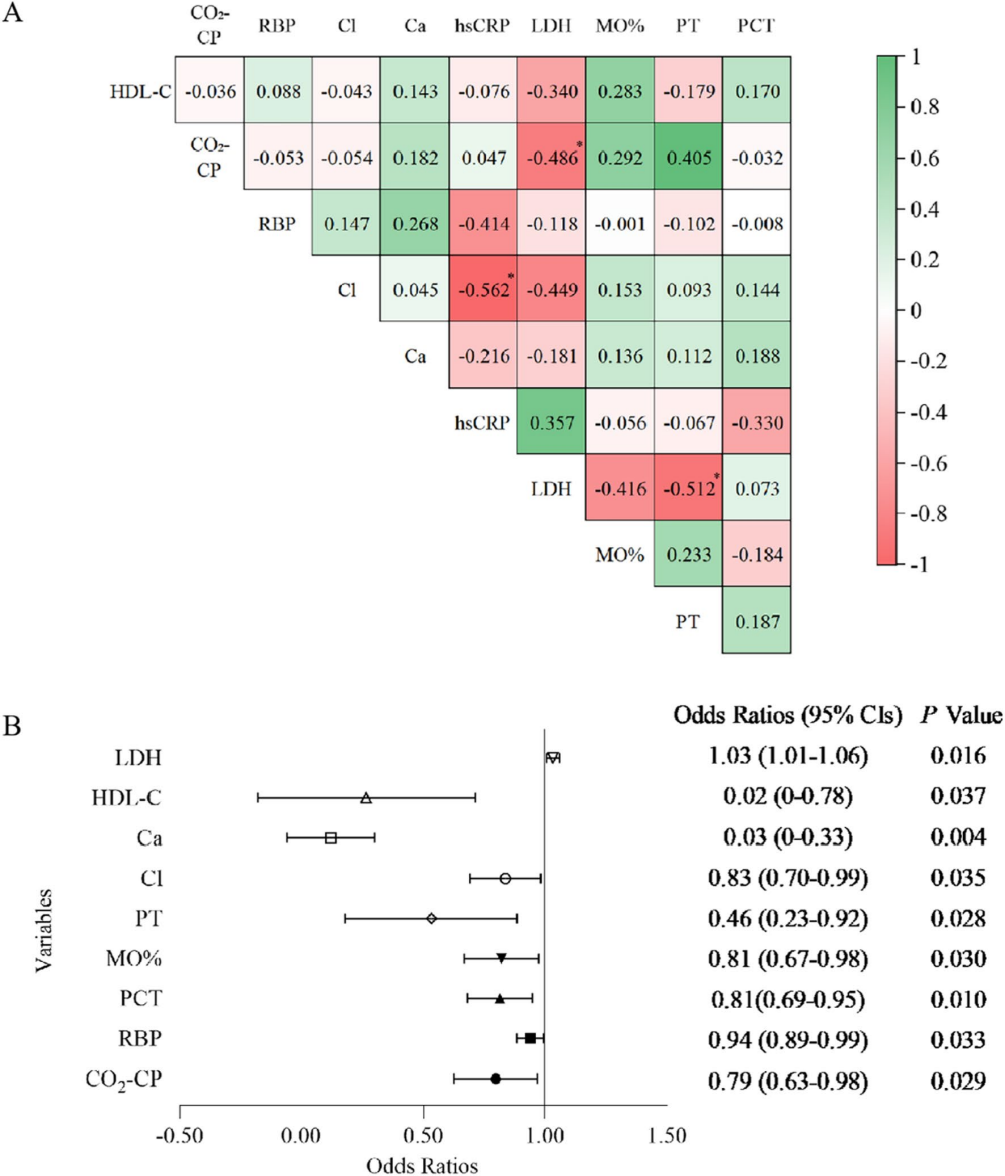
**a** Correlation heat map of 10 significantly different non liver function indicators in the training set. **b** Forest plot of laboratory indicators based on the univariate logistic regression analysis. *Significant correlation between the two indicators, *P* < 0.001. CO_2_-CP, Carbon dioxide combining power, mmol/L; RBP, Retinol-binding protein, mg/L; PCT, Plateletcrit, ‱; MO%, Monocyte percentage, %; PT, Prothrombin time, s; Cl, Chloride, mmol/L; Ca, Calcium, mg/dL; HDL-C, High-density lipoprotein cholesterol, mmol/L; hsCRP, High-sensitivity C-reactive protein, mg/L; LDH, Lactate dehydrogenase, U/L

**Fig. 2:**
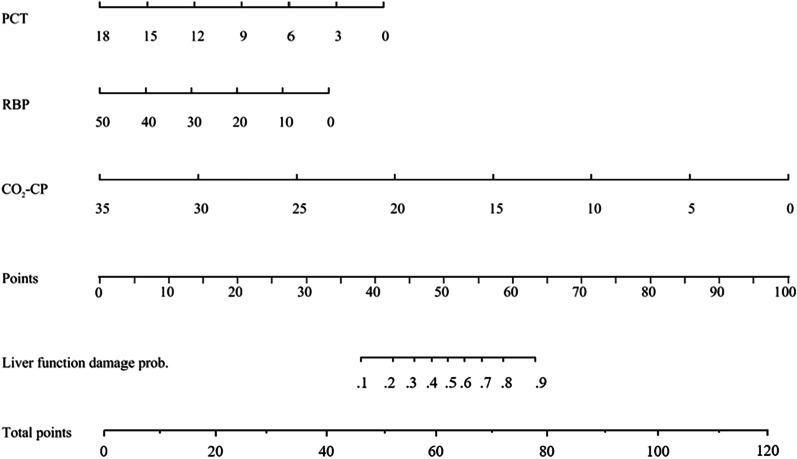
Nomogram to illustrate how PCT, RBP, and CO_2_-CP on admission are related to liver function damage. CO_2_-CP, Carbon dioxide combining power, mmol/L; RBP, Retinol-binding protein, mg/L; PCT, Plateletcrit, ‱

**Fig. 3:**
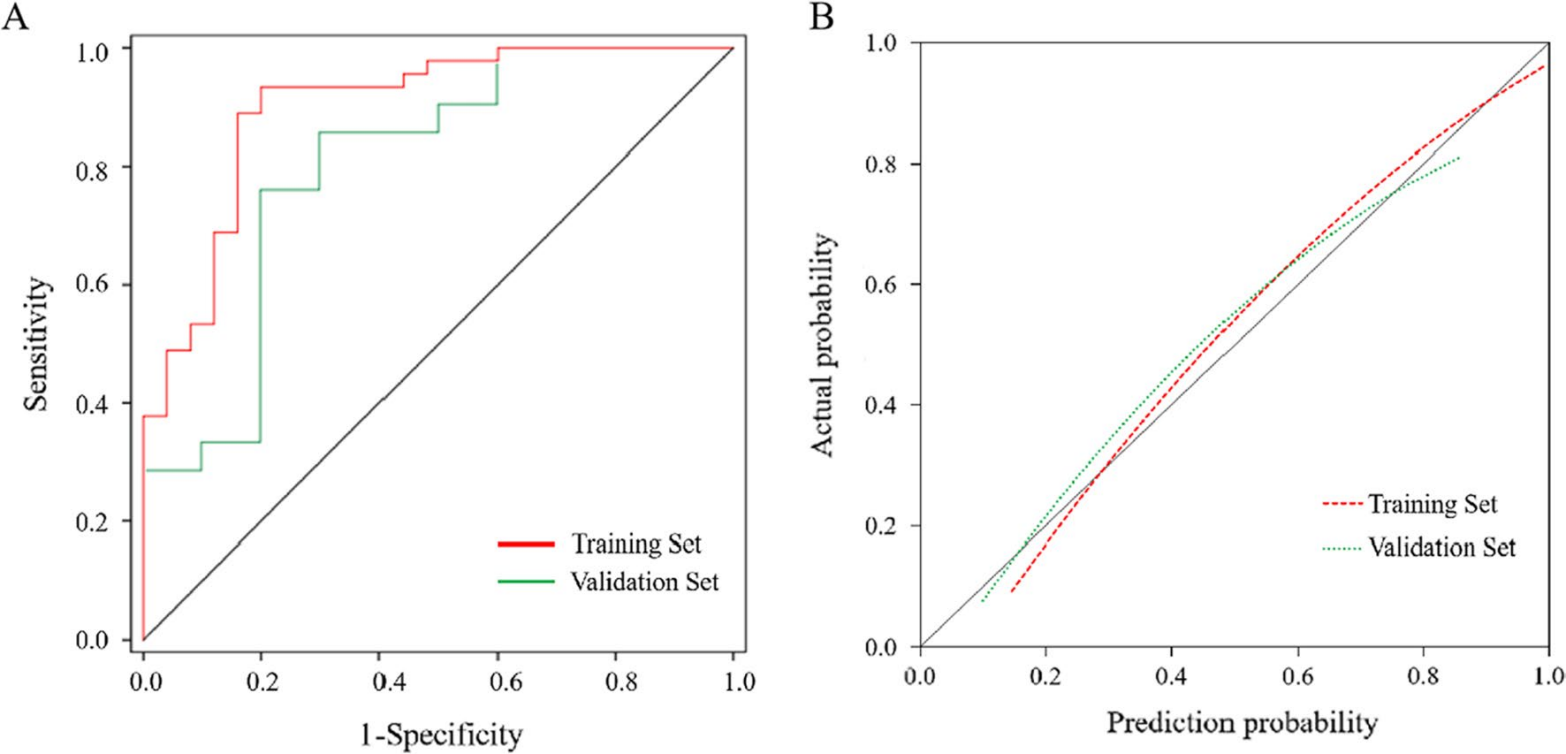
**a** Receiver operating characteristic curve. Training set area under the curve: 0.899, validation set area under the curve: 0.800. **b** Calibration curve

**Fig. 4:**
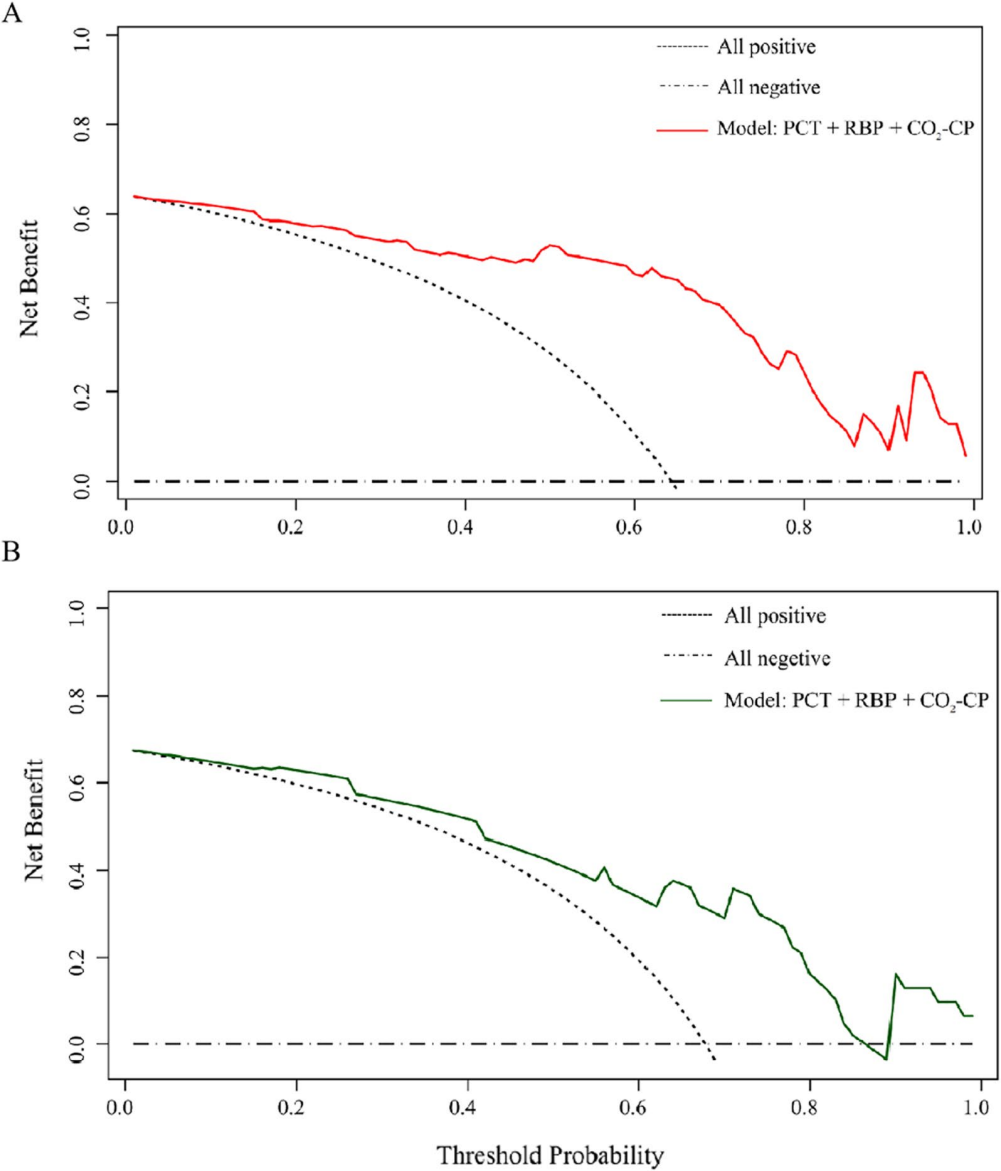
Decision curve analysis. **a** Training set. **b** Validation set. CO_2_-CP, Carbon dioxide combining power, mmol/L; RBP, Retinol-binding protein, mg/L; PCT, Plateletcrit, ‱

Spearman’s correlation coefficient showed significant correlations between hsCRP and Cl (γ =  − 0.562), LDH and PT (γ =  − 0.512), and LDH and CO_2_-CP (γ =  − 0.486) (*P* < 0.001 for all) (Fig. 1a). The univariate logistic regression analysis showed that CO_2_-CP, RBP, PCT, MO%, PT, Cl, Ca, HDL-C, and LDH were significantly different between the liver function damage group and the normal liver function group (*P* < 0.05 for all) (Fig. 1b). LDH and Cl were excluded in subsequent calculations to avoid multicollinearity bias in the multifactor analysis. The multivariate logistic regression analysis included CO_2_-CP, RBP, PCT, MO%, PT, Ca, HDL-C, and hsCRP. The results of the backward linear regression method showed that PCT (OR 0.73, 95% CI 0.62–0.86, *P* < 0.001), RBP (OR 0.91, 95% CI 0.86–0.97, *P* = 0.002), and CO_2_-CP (OR 0.67, 95% CI 0.48–0.95, *P* = 0.024) could jointly predict liver function damage in moderate cases, with a specificity of 80.0% and a sensitivity of 93.3%. A nomogram of the model was generated. Each variable was assigned a score, and the individual scores were summed to calculate a total score. The total score reflected each patient’s probability of liver function damage (Fig. 2). The ROC curve showed that the AUC in the training set was 0.899 (95% CI 0.820–0.977, *P* < 0.05), and the AUC in the validation set was 0.800 (95% CI 0.620–0.980, *P* < 0.05) (Fig. 3a). According to the Hosmer–Lemeshow goodness-of-fit test results in the calibration curve, in the training set, *P* = 0.59 > 0.05, and in the validation set, *P* = 0.19 > 0.05 (Fig. 3b). The DCA model had significant net benefits in training and validation sets (Fig. 4).

## Discussion

At present, the number of COVID-19 confirmed cases and deaths is still rising. Studies have shown that liver function damage in patients with moderate COVID-19 is associated with disease mortality [16], but few studies have analyzed the factors related to liver function damage. Liver function damage means that the patient’s detoxification, synthesis, and metabolic functions are reduced, so early detection and treatment of liver function damage are very important. This study included data from the first laboratory examination of 101 patients with moderate COVID-19 after admission to establish an early liver function prediction model.

The results of multivariate logistic regression analysis were influenced by the characteristics of the subjects [17]. Because of the imbalance in the study’s grouping population, there may be differences in their attributes (such as gender, age), between the two groups. In this study, all the patients were moderate COVID-19. After grouping in the training set, the gender ratio between groups tended to 1:1, and there was no significant age difference between the two groups. Therefore, the deviation caused by the research subjects could be ignored in this study. Second, the significant correlation among the research factors may bring bias to the research results [18]. In this study, Spearman’s correlation analyzed the correlation of the indexes. It eliminated the significantly related indexes to prevent other causes or confounders from affecting the study results.

In this study, the prediction model, which combined PCT, RBP, and CO_2_-CP to assess liver function damage in moderate cases, was established by logistic regression. PCT is the volume proportion of platelets in the blood and is used to monitor various liver diseases [19, 20], but its mechanism of action remains unclear. Studies have shown that reduced PCT can diagnose alcoholic cirrhosis [21] and predict liver fibrosis in patients with chronic hepatitis C [22]. However, one study [23] suggested that the PCT level in patients with non-alcoholic steatohepatitis is significantly higher compared with healthy controls. This study is consistent with former studies, which indicate that a lower PCT level can predict liver function damage in patients with moderate COVID-19. Platelets are involved in the body’s coagulation process, and abnormal PCT levels can indicate coagulation dysfunction, suggesting coagulopathy in patients with COVID-19 is may be related to liver function damage [24]. In addition, liver disease can cause kidney damage, and kidney damage can aggravate liver injury. There are close physiological and pathological relationships between the kidneys and liver [25].

RBP and CO_2_-CP are renal function monitoring and treatment [26, 27]. This study shows essential value in predicting liver function damage in patients with moderate COVID-19. RBP is a vitamin transporter in the blood. It is mainly synthesized in the liver and is widely distributed in body fluids. A previous study has shown that RBP4 levels are closely related to liver and kidney function in children with obesity [28]. Ito et al. [29] suggested that RBP can be used as a screening indicator for liver function damage, consistent with the results. As an indicator of renal function, CO_2_-CP has not been studied concerning liver function damage. This study suggested that the reduction in CO_2_-CP was related to liver function damage in patients with moderate COVID-19, providing a new idea for clinical research on the mechanism of liver function damage.

Zhang et al. [30] found that the male sex, a high D-Dimer concentration, and a high NE% were associated with liver function damage in patients with COVID-19. Moreover, Xie et al. [31] found that WBC count, neutrophil count, hsCRP, and chest computed tomography score were higher in patients with liver function damage than patients with normal liver function. However, these observations were not reflected in the present study. The possible reasons for these differences are as follows. First, there were differences between the study populations. This study only included patients with moderate COVID-19; patients with mild, severe, or critical COVID-19 were not included. Second, there were differences among study areas. This study was carried out in Jilin, China, and the above studies were all carried out in Hubei, China. Jilin province was one of the least affected areas in China, and the severity of the disease was lower than the severity of the illness in Hubei.

The patient received different drug treatments, such as antiviral, anti-inflammatory, and traditional Chinese medicine. The study results did not rule out the effects of some drugs on liver function. However, the predictive model was established in this study using data from the first laboratory test at admission, when most patients had not yet received drug treatment. Therefore, the effect of drugs on liver function is negligible. This study evaluated the model’s discrimination, calibration, and clinical usefulness by ROC curve analysis, calibration curve analysis, and DCA. The model performed well in all aspects. Thus, we believe that the model can be used in daily clinical work to help doctors determine liver function damage in patients with moderate COVID-19.

The limitations of this study are as follows. First, the small sample size of this study may have affected the statistical significance of the data. Second, this study only verified the prediction model internally, and its practicability needs to be verified using external data. Third, the data used in this study resulted from each patient’s first laboratory examination after admission, not the laboratory examination results at the symptomatic onset. If patients did not visit a doctor as soon as symptoms were detected, data at the symptomatic beginning would not be available.

## Conclusions

This study used logistic regression to establish a prediction model for liver function damage in patients with moderate COVID-19. The model’s discrimination, calibration, and clinical usefulness were evaluated by ROC curve analysis, calibration curve analysis, and DCA. PCT, RBP, and CO_2_-CP can be used as accurate biomarkers to predict liver function in patients with moderate COVID-19 at admission. By combining these three factors, the model can accurately predict liver function damage and liver disease progression in moderate cases.

## Data Availability

The datasets used and/or analysed during the current study are available from the corresponding author on reasonable request.

## Abbreviations

COVID-19: Corona virus disease 2019
ROC: Receiver operating characteristic
AUC: Area under curve
OR: Odds ratio
CI: Confidence interval
DCA: Decision curve analysis
CO_2_-CP: Carbon dioxide combining power
RBP: Retinol-binding protein
PCT: Plateletcrit
WBC: White blood cell
NE%: Neutrophil percentage
MO%: Monocyte percentage
PT: Prothrombin time
Cl: Chloride
Ca: Calcium
HDL-C: High-density lipoprotein cholesterol
hsCRP: High-sensitivity C-reactive protein
LDH: Lactate dehydrogenase
